# A new promising biological agent for refractory lupus nephritis: Telitacicept

**DOI:** 10.12669/pjms.39.4.7542

**Published:** 2023

**Authors:** Shuangyan Liu, Xiangyue Yu, Xiaodong Li

**Affiliations:** 1 Shuangyan Liu, Graduate School of Hebei Medical University, Shijiazhuang 050017, Hebei, China; 2 Xiangyue Yu, Department of Nephrology, Baoding No. 1 Central Hospital of Hebei Medical University, Baoding, Hebei, China; 3 Xiaodong Li, Department of Nephrology, Baoding No. 1 Central Hospital of Hebei Medical University, Baoding, Hebei, China

Systemic lupus erythematosus (SLE) is a common autoimmune system disease characterized by high expression of auto-antibodies and multi-system involvement. Lupus nephritis (LN) is the most common serious complication of SLE. Refractory LN, not response to mycophenolate mofetil or cyclophosphamide, is often difficult to treat. Massive proteinuria and IV or IV + V types of LN are main causes of refractory LN.^1^ Telitacicept is a new dual B lymphocyte stimulator and proliferation inducing ligand inhibitor that may effectively block proliferation of B lymphocytes.^2^ It could be applicable for B lymphocyte-mediated diseases, such as SLE, LN, which was approved for the treatment of adult patients in China on March 9, 2021.^3^ Herein, we present a young patient with refractory LN, who was safely and effectively treated with telitacicept.

A 31-year-old woman was admitted to our hospital with edema and oliguria. She was diagnosed with SLE and LN,9 years ago and had received long-term standard therapies, including sufficient glucocorticoids and cyclophosphamide, following tacrolimus, mycophenolate mofetil and Chinese herbal medicine. She refused renal biopsy and her 24hours urinary proteins did not improved despite the therapy. She complained about her obesity, anxiety and insomnia, and are discontinued glucocorticoids against medical advice. Physical examinations at admission showed severe edema in both lower extremities. Laboratory examinations results in our hospital showed in Table-I which suggested her complications with acute kidney injury. Chest computed tomography showed moderate amount of bilateral pleural effusion. Echocardiography showed a mild pericardial effusion. The patient was diagnosed with SLE and refractory LN. Three days later, she developed high fever with vomiting and diarrhea, and was diagnosed with acute gastroenteritis. After antibiotic therapy for a week, her gastroenteritis relieved. The patient received renal biopsy showing LN type IV+V. Then she received administration of telitacicept (skin-pop, 160 mg once a week), methylprednisolone (20 mg once a day) and hydroxychloroquine (0.1g twice a day). She was followed up every month after discharge. Subsequent re-examination suggested a significant reduction in 24hours urinary proteins, an increase in plasma albumin level, and a remarkable improvement in her renal function and general condition. She is currently undergoing regular long-term recovery, during which her condition is stable.

**Table-I:** Lab examination in our hospital.

Variable	Normal Range	Day 1	Day 7	Month 1	Month 2	Month 3
WBC	4-10 (×10^9^/L)	2.54	5.39	7.37	9.97	10.61
RBC	3.5-5(×10^9^/L)	2.50	2.61	3.45	4.75	5.53
Hb	110-150(g/L)	71.0	74.0	98.0	125.0	130.0
Lymphocyte	1.2-4.8(×10^9^/L)	1.05	1.46	2.00	3.62	3.64
Neutrophil	1.5-8.0(×10^9^/L)	1.25	3.34	4.84	5.49	6.09
Alb	40-55(g/L)	12.1	11.6	18.0	17.3	24.9
24hUpro	0-150mg	9.85	11.23	8.56	6.73	5.96
Serum creatinine	57-97mmol/L	148.6	225.3	165.4	128.6	95.1
Ig A	8.6-17.4mmol/L	19.6	18.5	16.3	14.2	7.6
Ig G	0.56-1.7mmol/L	2.53	2.78	1.89	0.88	0.45
Ig M	0.3-2.2 mmol/L	2.18	2.37	1.76	1.24	0.33
C3	0.7-1.4mmol/L	0.36	0.45	0.52	0.83	0.91
C4	0.10-0.40mmol/L	0.08	0.07	0.09	0.12	0.24
ANA	<1:100	1000-3200	/	<1000	<1000	<320
Anti-Sm	-	+++	/	++	++	+
Anti-dsDNA	-	-	/	-	-	-
Anti-Ro-52	-	++	/	++	+	+
CRP	0-8(mmol/L)	24.32	78.6	/	/	10.50
ESR	0-15(mm/h)	122	/	/	/	29

WBC (white blood cell), RBC (Red blood cells), Hb(hemoglobin), Alb(albumin), 24hUpro (24 hours urine proteins 0-1.5g), Immune globulin (Ig), ANA (anti-nuclear antibody), CRP (C-reactive protein), ESR (erythrocyte sedimentation rate).

The patient had received a long-term standardized treatment but did not achieve remission. She could not tolerate glucocorticoid. Hence, we tried telitacicept as a new therapy. After three months of treatment with telitacicept, her renal function markedly improved. The changes of her urine proteins, renal function and immunological test are shown in Table-I. Telitacicept has been used as the therapy for SLE in China, especially in LN, which may significantly decrease the dosage of glucocorticoids and immunosuppressive.^4^ Our experience shows that Telitacicept combined with standard therapy are effective in treating Chinese patients with moderate or severe SLE.^5^ However, its long-term efficacy and safety must be further observed and investigated. LN is the most common manifestation of SLE which can lead to kidney failure among a subset of patients. Kidney involvement has been associated with higher mortality in SLE patients. Persistent activity or frequent flares could lead to the progressive end-organ damage. Therefore, it is important to select an effective treatment and achieve remission at an early stage in SLE. Furthermore, the ultimate goal of treating LN is to protect kidney function, reduce the mortality with SLE and kidney failure, and decrease treatment -associated side effects.^6^

Although the results of Phase-III in clinical trials of telitacicept have not yet been announced, the findings of its Phase-I and II clinical trials of it have shown the effectiveness and safety for SLE patients.^5^ Our case suggests that telitacicept may be an effective biological agent to treat refractory LN in the near future. However, the current research data are limited, large-scale clinical trials should continue to be carried out for further verification.

## Authors’ contributions:

**XL:** Study design and manuscript revision.

**SL:** Manuscript writing.

**XY:** Formulating patient’s treatment.

All authors contributed to the article and approved the submitted version.

## References

[1] Kidney Disease:Improving Global Outcomes (KDIGO) Glomerular Diseases Work Group (2021). KDIGO2021 Clinical Practice Guideline for the Management of Glomerular Diseases. Kidney Int.

[2] Shi F, Xue R, Zhou X, Shen P, Wang S, Yang Y (2021). Telitacicept as a BLyS/APRIL dual inhibitor for autoimmune disease. Immunopharmacol Immunotoxicol.

[3] Dhillon S (2021). Telitacicept:First Approval. Drugs.

[4] Chen R, Fu R, Lin Z, Huang C, Huang W (2022). The efficacy and safety oftelitacicept for the treatment of systemic lupus erythematosus:a real life observational study. Lupus.

[5] Fan Y, Gao D, Zhang Z (2022). Telitacicept, a novel humanized, recombinant TACI-Fc fusion protein, for the treatment of systemic lupus erythematosus. Drugs Today (Barc).

[6] Mahajan A, Amelio J, Gairy K, Kaur G, Levy RA, Roth D (2020). Systemic lupus erythematosus, lupus nephritis and end-stage renal disease:a pragmatic review mapping disease severity and progression. Lupus.

